# Significance of *LRFN4* in prognosis and tumor microenvironment of lung adenocarcinoma

**DOI:** 10.3389/fphar.2025.1540636

**Published:** 2025-02-25

**Authors:** Nana Wang, Shuming Cao, Xiaofeng Tan, Meirong Liu

**Affiliations:** ^1^ Department of General Internal Medicine, Tianjin Hospital, Tianjin, China; ^2^ Department of Hand Surgery, Tianjin Hospital, Tianjin, China

**Keywords:** *LRFN4*, lung adenocarcinoma, E2F1, E2F3, immune landscape, drug sensitivity

## Abstract

**Background:**

*LRFN4* is expressed in various tumors and leukemia cell lines. This study aims to explore the effect of *LRFN4* in lung adenocarcinoma (LUAD).

**Methods:**

The data on LUAD patients were collected from the Cancer Genome Atlas and Gene Expression Omnibus database. The expression of *LRFN4* in LUAD and LUAD cell lines was analyzed via differential gene analysis, qRT-PCR assay, and Western blotting assay. The correlation of *LRFN4* expression with the onset of LUAD were calculated using Pearson correlation analysis. The transcription factors correlated with *LRFN4* expression were screened by differential expression analysis and Pearson correlation analysis. Moreover, the effect of *LRFN4* on the immune landscape of LUAD was analyzed using CIBERSORT algorithm. The GDSC and CTRP databases were used to analyze the drug sensitivity of hub gene.

**Results:**

*LRFN4* was highly expressed in LUAD patients and cells, and *LRFN4* expression was correlated with metastasis, pathological stages, and age of LUAD patients. The transcription factors E2F1 and E2F3 could regulate *LRFN4* expression by binding upstream of *LRFN4*. The 8 immune cell infiltration levels were differential between *LRFN4* ^high^ and *LRFN4* ^low^ patients. The ESTIMATEScore and ImmuneScore levels were decreased, the TumorPurity level was elevated, and 6 immune checkpoint expressions were increased in LRFN4^high^ patients. Moreover, LRFN4^high^ patients had inferior prognosis. The mutation rate of TP53, TTN, and MUC6 and the level of TMB were increased in *LRFN4* ^high^ patients. The expressions of TCF3, E2F1, E2F3, and *LRFN4* were correlated with the IC50 of multiple drugs.

**Conclusion:**

*LRFN4* may serve as a novel prognostic biomarker for LUAD, shows specific overexpression in LUAD and may be a potential therapeutic target for LUAD patients.

## 1 Introduction

Lung adenocarcinoma (LUAD) is the most frequent form of primary lung cancer, accounting for more than 40% of these cases, and its prevalence is rising (Zhao et al., 2023; Chen et al., 2024; Wei et al., 2024). The major symptoms of LUAD patients are shortness of breath and a persistent cough. LUAD is divided into several subtypes, including adenocarcinoma *in situ* (AIS), minimally invasive adenocarcinoma (MIA), invasive adenocarcinoma, and variants of invasive adenocarcinoma (Liu et al., 2019b). However, the therapies for LUAD include many approaches, such as radiotherapy, surgical resection, chemotherapy, immunotherapy, and radiotherapy (Hirsch et al., 2017). As LUAD often metastasizes by the time it’s diagnosed, identifying patients in the early stages has been a great challenge in clinical practice (Lin et al., 2021b). In addition, LUAD cells can rapidly develop drug resistance after initial therapy and are often untreatable with chemotherapeutic drugs (Min et al., 2022; Ascenzi et al., 2024). The 5-year overall survival rate of LUAD remains less than 20% (Lin et al., 2016). Thus, it is necessary to explore novel biomarkers to guide the prognosis of LUAD patients.

Leucine-rich repeat and fibronectin type III domain-containing proteins (LRFN), also known as synaptic adhesion-like molecules (SALM), are a family of leucinerich repeat (LRR)-containing synaptic cell adhesion molecules (Lie et al., 2018; Gu et al., 2023). This family consists of five know members: LRFN1/SALM2, LRFN2/SALM1, LRFN3/SALM4, LRFN4/SALM3, and LRFN5/SALM5 (Lie et al., 2018). It has been demonstrated that LRFN plays important roles in neurite outgrowth and synapse formation (Karki et al., 2020; Xu et al., 2023). A study that evaluated all known members of the LRFN family has discovered that only LRFN4 and LRFN5 were capable of initiating presynaptic differentiation in axons (Mah et al., 2010). Recently, *LRFN4* has been found to be expressed in a variety of tumors and leukemia cell lines (Konakahara et al., 2011). This study has also reported that in the monocytic cell line THP-1 and in primary monocytes, *LRFN4* expression is elevated following macrophage differentiation, and *LRFN4* signaling could regulate both transendothelial migration and elongation of THP-1 cells by actin cytoskeleton reorganization (Konakahara et al., 2011). Zheng and colleagues have documented that in colorectal cancer (CRC), *LRFN4* expression was tightly correlated with tumor location and TNM staging (Zheng et al., 2020). *LRFN4* is upregulated in clinical gastric cancer cells and fibroblasts, and a high level of *LRFN4* is found to be substantially linked with tumor invasive features and shorter survival rate of patients (Liu et al., 2019a), and *LRFN4* overexpression is associated with higher risk of gastric cancer (Huang et al., 2021). In addition, *LRFN4* is a risk gene for ovarian cancer (Li et al., 2021) and a prognostic biomarker for stomach adenocarcinoma (Han et al., 2021). However, to the best of our knowledge, the role of *LRFN4* in LUAD has rarely been reported.

Thus, the purpose of this work is to explore the effect of *LRFN4* in LUAD, and to investigate the molecular mechanism of *LRFN4* in regulating the progression of LUAD. This study is the first to report the role of *LRFN4* in the prognosis and immune microenvironment of LUAD patients, and is expected to provide a reference for the future development of new LUAD diagnosis and treatment strategies.

## 2 Materials and methods

### 2.1 Data collection

The mRNA expression profiles of 585 samples, including 525 LUAD and 60 normal samples, were obtained from The Cancer Genome Atlas (TCGA, https://tcga-data.nci.nih.gov/tcga/) database. Among 585 samples, 501 samples contained complete survival information (Table 1). In addition, the GSE116959, GSE19188 and GSE68465 datasets were downloaded from Gene Expression Omnibus (GEO, https://www.ncbi.nlm.nih.gov/geo/) database. GSE116959 included 57 LUAD samples and 11 normal samples, GSE19188 contained 91 LUAD samples and 65 normal samples, among which 82 samples had valid survival information. GSE68465 included 442 LUAD samples with complete and valid survival information. The data of three datasets (GSE116959, GSE19188 and GSE68465) were obtained using Agilent-039494 SurePrint G3 Human GE v2 8 × 60K Microarray 039,381, Affymetrix Human Genome U133 Plus 2.0 Array, and Affymetrix Human Genome U133A Array platforms, respectively.

**TABLE 1:** Clinicopathological characteristics of Osteosarcoma patients from TCGA-LUAD database.

Characteristics		Patients (N = 501)	
		NO.	%
Gender	Female	271	54.09%
	Male	230	45.91%
Age	≤66 (Median)	259	51.70%
	>66 (Median)	242	48.30%
Grade	I	269	53.69%
	II	119	23.75%
	III	80	15.97%
	IV	25	4.99%
	Unknown	8	1.60%
Survival Time	Long (>5 years)	251	50.10%
	Short (<5 years)	52	10.38%
OS status	Dead	182	36.33%
	Alive	319	63.67%
Radiation	Yes	416	83.03%
	No	71	14.17%
	Unknown	14	2.79%
Tobacco	Yes	58	11.58%
	No	361	72.06%
	Unknown	82	16.37%

### 2.2 Differential gene analysis

The differential gene analysis was carried out between the two groups using the “limma” (Ritchie et al., 2015) function package in R (version 4.2.1, the same below). The |Log2FC| > 1 and P.adjust <0.05 were used to screen the differentially expressed genes (DEGs).

### 2.3 Cell collection and culture

The human lung epithelial cell line BEAS-2B (SCSP-5067) and human LUAD cell lines NCI-H1395 (NCI-H1395), NCI-H1975 (SCSP-597), and NCI-H441 (SCSP-5239) were obtained from the cell bank of the Type Culture Collection Committee of the Chinese Academy of Sciences (Shanghai, China). These cell lines were cultured in RPMI-1640 medium (11,875,085, GIBCO, Carlsbad, CA) supplemented with 10% fetal bovine serum (FBS, A5670701, GIBCO, Carlsbad, CA) and maintained in a chamber at 37°C with 5% CO_2_.

### 2.4 qRT-PCR assay

The cells were utilized for RNA extraction with the TRIzol reagent (Invitrogen, Waltham, Massachusetts, USA). Following this, the RNA underwent conversion into cDNA utilizing the HiScript IV RT SuperMix for qPCR (+gDNA wiper) kit (Vazyme, R423-01, Nanjing, China). AceQ Universal SYBR qPCR Master Mix (Vazyme, Q511-02, Nanjing, China) was employed for qRT-PCR as per the manufacturer’s guidelines. Table 2 displayed the primer sequences. GADPH served as the internal reference, with each sample undergoing triplicate runs. mRNA expression levels were determined using the 2^−ΔΔCT^ calculation.

**TABLE 2:** List of the qPCR primers used in this study.

Genes	Forward primer (5′-3′)	Reverse primer (5′-3′)
LRFN4	GAC​CAT​AAC​CTT​ATT​GAC​GCA​CT	CAT​CAC​GCC​CAC​GAG​AGA​AA
GAPDH	ACA​ACT​TTG​GTA​TCG​TGG​AAG​G	GCC​ATC​ACG​CCA​CAG​TTT​C

### 2.5 Western blotting assay

Total protein was extracted from cells using the RIPA buffer (Solarbio, China) according to the manufacturer’s guidelines. Western blotting assay was performed essentially as described previously (Wang et al., 2024). The primary antibodies used in this study included Anti-LRFN4 (Abcam, ab106369, USA) and beta Tubulin Polyclonal Antibody (Proteintech, 10068-1-AP, China). The secondary antibody was HRP-conjugated Affinipure Goat Anti-Rabbit IgG (H + L) (SA00001-2, Proteintech). The internal reference used in this study was Tubulin. The Western blot signal intensities were quantified using Image software.

### 2.6 Gene set enrichment analysis (GSEA)

In TCGA cohort, the patients were split into *LRFN4* ^high^ and *LRFN4* ^low^ groups according to the median value of *LRFN4* expression. The DEGs between these two groups were obtained and were then subjected to GSEA using R language function package “clusterprofiler” (Yu et al., 2012). The significantly enriched pathways were screened by |NES| >1 and P < 0.05.

### 2.7 Survival analysis

The overall survival rate of different groups was estimated using the R language “survival” and “survminer” packages (https://CRAN.R-project.org/package=survival). The significance of differences in survival rate between different groups was tested using the log-rank. The multivariate Cox regression model was used to analyze whether the target gene could predict the survival of LUAD patients independently of other factors.

### 2.8 Immune cell infiltration

The CIBERSORT software (Newman et al., 2015) was applied to calculate the relative proportions of the 22 immune cells in the samples. CIBERSORT can describe the composition of immune infiltrating cells using the 547 preset barcode genes in deconvolution algorithm, based on the gene expression matrix. The immune score of samples was calculated using the “estimate” function package (https://R-Forge.R-project.org/projects/estimate/). Moreover, the tumor immune dysfunction and ejection (TIDE, http://tide.dfei.harvard.edu/) score was used to evaluate tumor immunotherapy response.

### 2.9 Screening of transcription factors correlated with LRFN4 expression

In TCGA-LUAD mRNA cohort, the significantly differentially expressed transcription factors were selected using the DEGs between LUAD and normal samples according to LogFC >1 and P < 0.05. The correlation of transcription factors with *LRFN4* expression were calculated using Pearson correlation, and the transcription factors significantly correlated with *LRFN4* were screened according to P < 0.05 and Rho >0.5.

### 2.10 Prediction of transcription factor binding sites

The 1,000 bp sequence file upstream of *LRFN4* gene start site was downloaded from UCSC (http://genome.ucsc.edu/). The corresponding motif file for the transcription factor was downloaded from the JASPER database (https://jaspar.genereg.net/). The online tool FIMO (https://meme-suite.org/meme/tools/fimo) was then used to predict whether there is a transcription factor binding motif in the region upstream of *LRFN4* promoter.

### 2.11 Drug sensitivity analysis

The half-maximal inhibitory concentration (IC50) values of 265 small molecules from the Genomics of Drug Sensitivity in Cancer (GDSC) database in 860 cell lines, along with their corresponding mRNA levels, and those of 481 small molecules from the Cancer Therapeutics Response Portal (CTRP) database in 1,001 cell lines, along with their corresponding mRNA levels, were collected. Combined the mRNA expression and drug sensitivity data, the relationship between drug IC50 and gene mRNA expression was obtained using Pearson analysis. The P-values were adjusted by false discovery rate (FDR).

### 2.12 Statistical analysis

The Wilcoxon rank sum test was utilized to compare the expression differences of *LRFN4* in tumor and normal samples, as well as other clinicopathological characteristics. Univariate cox regression analysis was used to analyze the correlation between LRFN4 and other reported biomarkers and prognosis. The effects of the mRNA expression of *LRFN4* and clinicopathological characteristics on overall survival were determined through a multivariate Cox regression proportional hazards model. Statistical significance was considered present when the P < 0.05.

Additionally, the statistical analysis of all experimental data was performed with GraphPad Prism version 9.5.0, and the results are presented as the mean ± standard deviation (SD). For comparisons between two groups, an unpaired two-tailed t-test was employed, and a p-value of less than 0.05 was considered statistically significant.

## 3 Results

### 3.1 LRFN4 was highly expressed in LUAD patients, and LRFN4 was correlated with metastasis, pathological stages and age of LUAD

**FIGURE 1:**
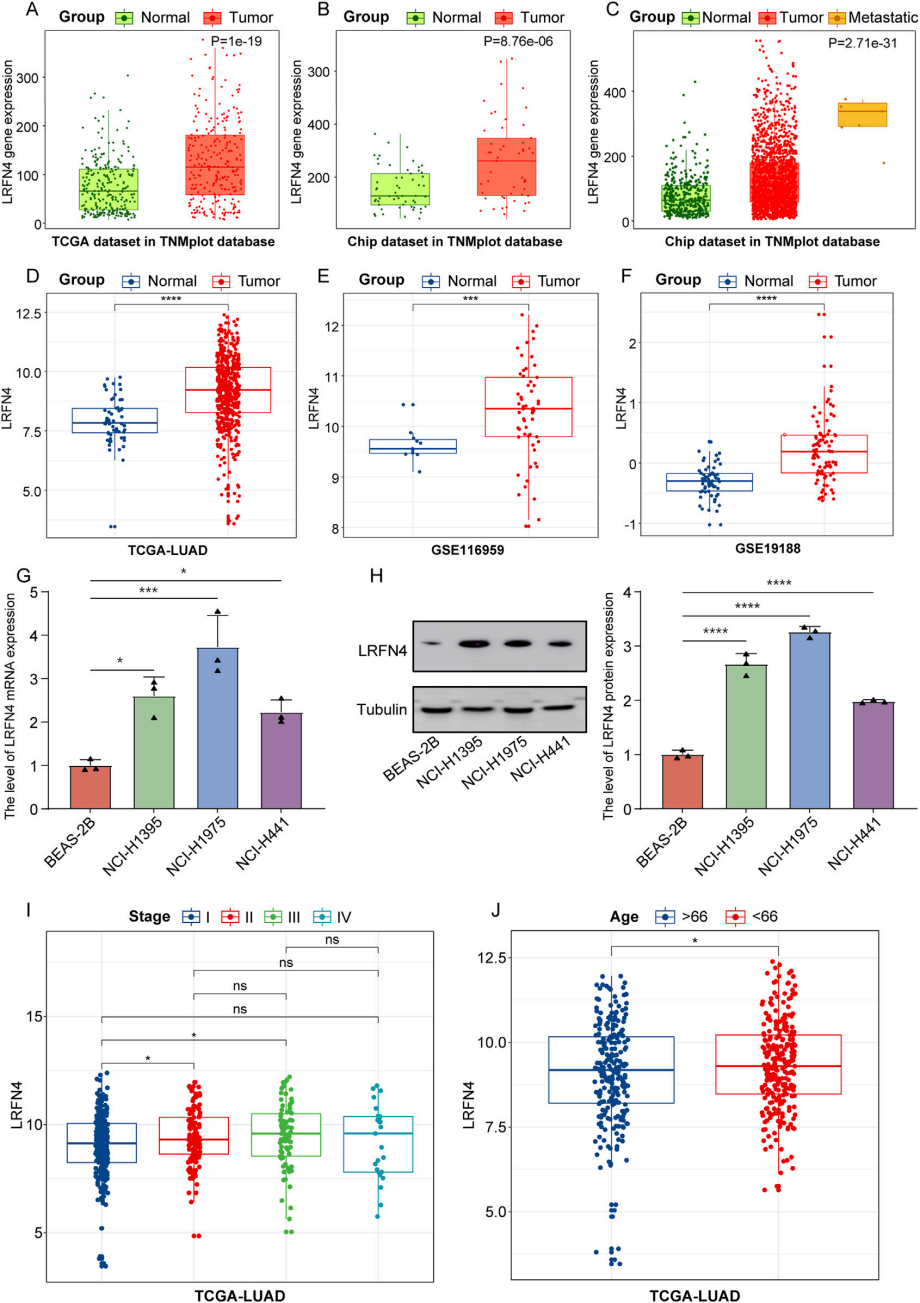
*LRFN4* was high expressed in LUAD patients, and *LRFN4* expression was correlated with metastasis, pathological stages and age of LUAD. **(A, B)** The expression of *LRFN4* in LUAD samples in both TCGA and Chip datasets in TNMplot database. **(C)** The expression of *LRFN4* in metastatic and non-metastatic LUAD samples in Chip datasets in TNMplot database. **(D–F)** The expression of *LRFN4* in LUAD samples in TCGA-LUAD, GSE116959, GSE19188 datasets, respectively. **(G)** The expression of *LRFN4* mRNA in the three LUAD cell lines was measured by qRT-PCR assay. **(H)** The expression of LRFN4 protein in the three LUAD cell lines was measured by Western blotting assay. **(I)** The expression of *LRFN4* in different pathological stages (I, II, III, IV) of LUAD in TCGA-LUAD cohort. **(J)** The correlation of *LRFN4* expression with age in TCGA-LUAD cohort *p < 0.05, ***p < 0.001, ****p < 0.0001, ns: no significant.

Firstly, we analyzed the expression of *LRFN4* in LUAD patients in TNMplot database (The entire database contains 56,938 samples, including 33,520 samples from 3180 gene chip-based studies (453 metastatic, 29,376 tumorous and 3691 normal samples), 11,010 samples from TCGA (394 metastatic, 9886 tumorous and 730 normal), 1193 samples from TARGET (1 metastatic, 1180 tumorous and 12 normal) and 11,215 normal samples from GTEx). We found that in the TNMplot database, *LRFN4* expression was significantly increased in LUAD samples compared to normal samples in both TCGA and Chip datasets (Figures 1A, B), and *LRFN4* upregulated in metastatic LUAD samples in Chip datasets (Figure 1C, metastatic vs non-metastatic, p < 0.001). As expected, the expression level of *LRFN4* in TCGA-LUAD and GEO cohorts were consistent with TNMplot database (Figures 1D–F). We also performed qRT-PCR and Western blotting assay verification, and the results showed that *LRFN4* expression trend in LUAD cells was consistent with the transcriptome result (Figures 1G, H).

Next, we analyzed the correlation of *LRFN4* expression with different pathological stages (I, II, III, IV) of LUAD in TCGA-LUAD cohort, and found that *LRFN4* expression was increased in stage II and III compared to stage I (Figure 1I) and was significantly higher in patients younger than 66 years compared to those older than 66 years (Figure 1J). Nevertheless, *LRFN4* expression was not remarkably differential between men and women (Supplementary Figure S1). In short, *LRFN4* was highly expressed in LUAD patients, and *LRFN4* probably contributed to the malignant progression of LUAD.

### 3.2 The pathways correlated with LRFN4 in LUAD

**FIGURE 2:**
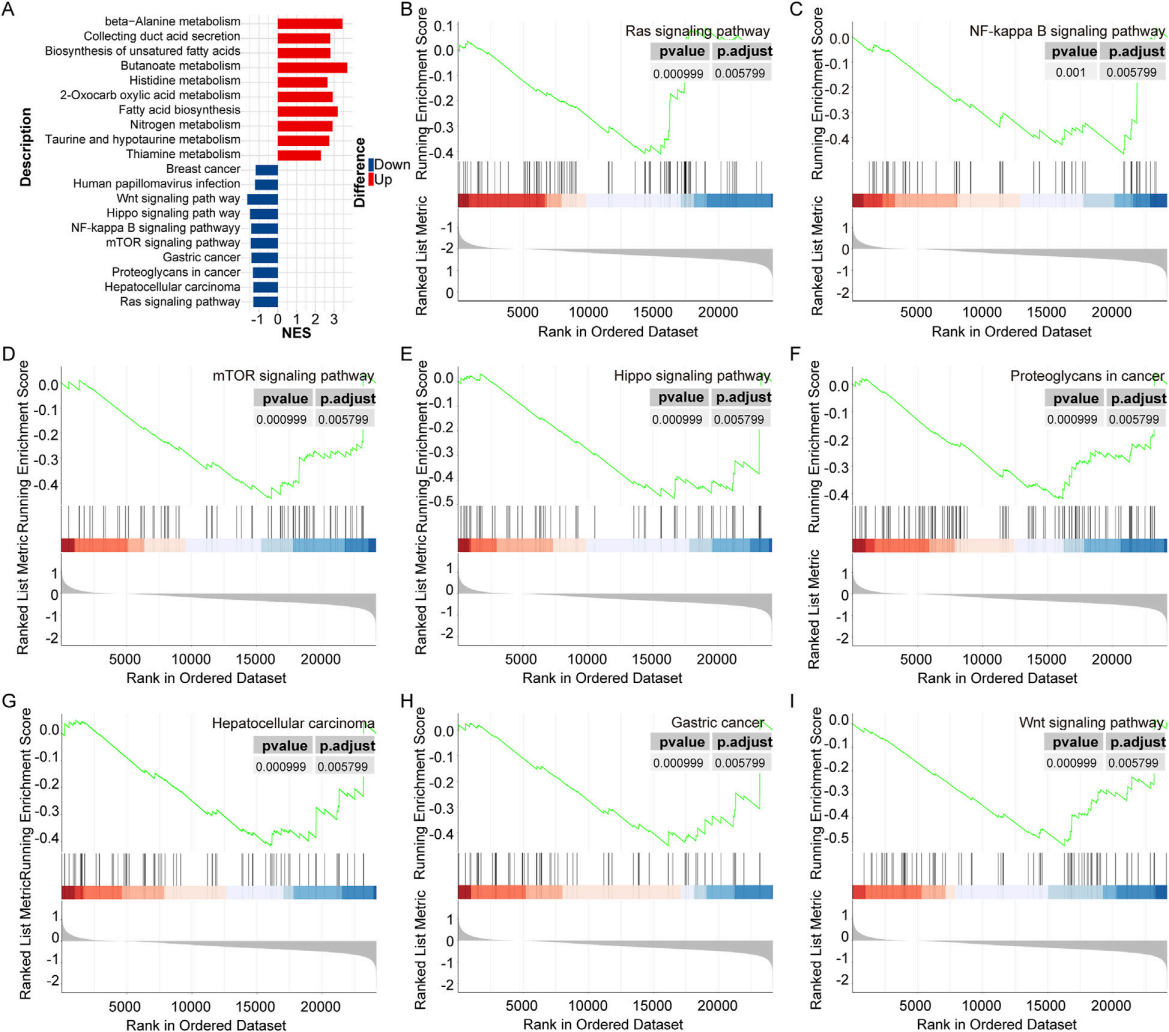
The pathways correlated with *LRFN4* in LUAD. **(A)** The top 10 significantly activated and inhibited pathways. **(B–I)** The Ras signaling pathway, Hepatocellular carcinoma, Proteoglycans in cancer, Gastric cancer, NF-kappa B signaling pathway, mTOR signaling pathway, Hippo signaling pathway and Wnt signaling pathway were correlated with cancer.

The GSEA results showed that a total of 19 and 86 signaling pathways were observably activated and inhibited in *LRFN4* ^high^ group compared to in *LRFN4* ^low^ group, respectively (Supplementary Table S1; Figure 2A (top 10 activated and inhibited terms)). Among which, the Ras signaling pathway, Hepatocellular carcinoma, Proteoglycans in cancer, Gastric cancer, NF-kappa B signaling pathway, mTOR signaling pathway, Hippo signaling pathway and Wnt signaling pathway were widely reported to correlate with various cancers (Figures 2B–I).

### 3.3 The transcription factors E2F1 and E2F3 could regulate LRFN4 expression by binding upstream of LRFN4

**FIGURE 3:**
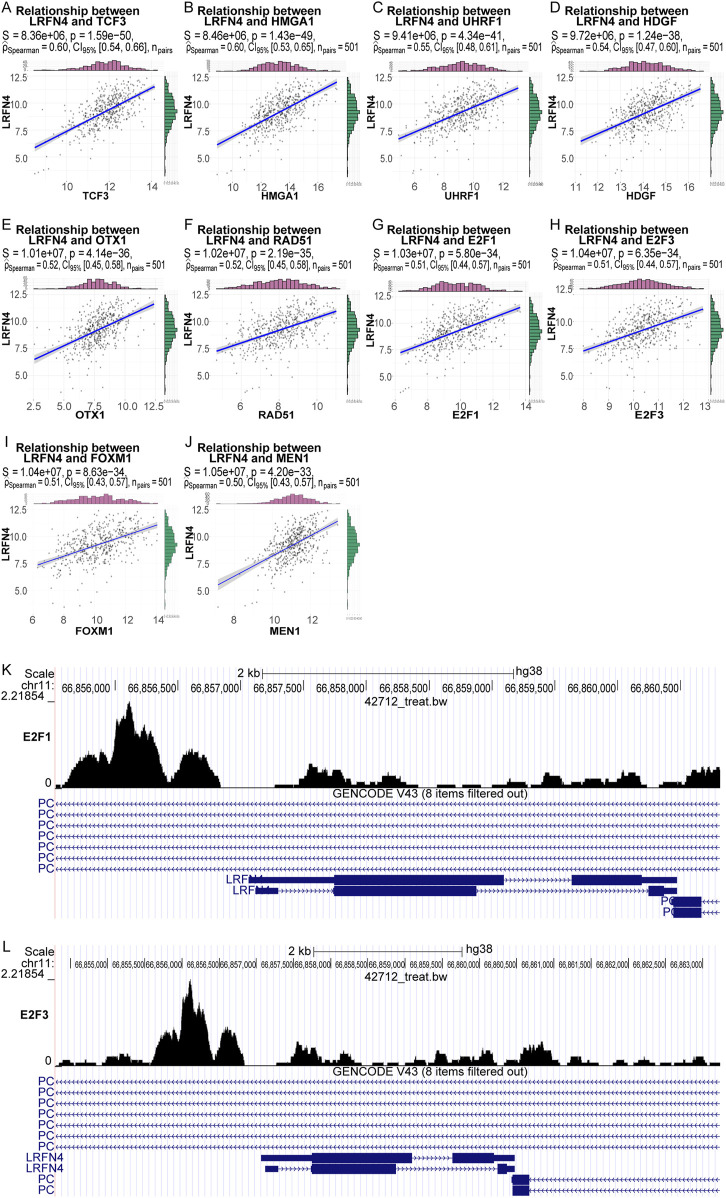
The transcription factors E2F1 and E2F3 could regulate LRFN4 expression by binding upstream of *LRFN4*. **(A–J)** The 10 transcription factors (TCF3, HMGA1, UHRF1, HDGF, OTX1. RAD51, E2F2, E2F3, FOXM1, MEN1) expression were dramatically positively correlated with *LRFN4* expression. **(K)** E2F1 had a distinct binding peak upstream of the LRFN4 gene in Breast-GSM2501567 dataset. **(L)** E2F3 exhibited a distinct binding peak upstream of the LRFN4 gene in Colon-GSM1239452 dataset.

We obtained 212 differentially expressed transcription factors between LUAD and normal samples. The Person correlation analysis showed that 10 transcription factors (TCF3, HMGA1, UHRF1, HDGF, OTX1. RAD51, E2F2, E2F3, FOXM1, MEN1) were dramatically positively correlated with *LRFN4* expression (Figures 3A–J). We discovered that in the about 369bp, 761bp, 844bp upstream of *LRFN4* promoter, there were binding sites for transcription factors TCF3 (MA0522.3.meme), E2F1 (MA0024.2.meme) and E2F3 (MA0469.1.meme), respectively (Supplementary Table S2). These results suggested that the transcription factors TCF3, E2F1 and E2F3 might regulate *LRFN4* expression by binding upstream of *LRFN4*.

Furthermore, in chip-seq database (http://cistrome.org/db/#/), we found that E2F1 and E2F3 had a distinct binding peak at upstream of *LRFN4* gene in Breast-GSM2501567 dataset (Gallenne et al., 2017) (Figure 3K, score = 2.693) and in Colon-GSM1239452 dataset (Yan et al., 2013) (Figure 3L, score = 1.856), respectively. Thus, transcription factors E2F1 and E2F3 could regulate *LRFN4* expression by binding at upstream of *LRFN4*.

### 3.4 LRFN4 might involve in affecting the immune landscape of LUAD

**FIGURE 4:**
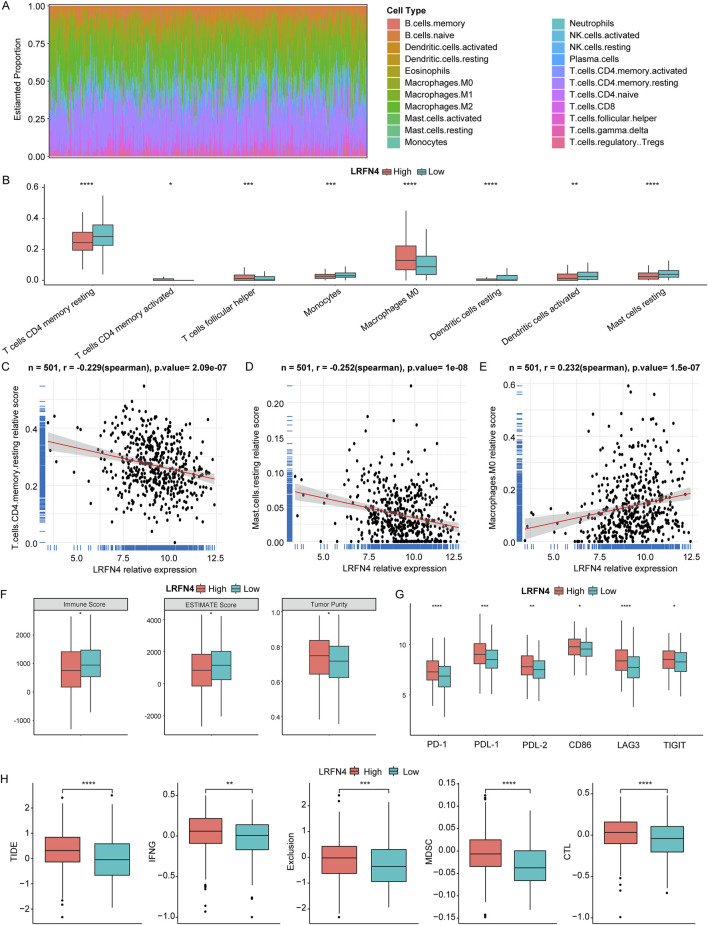
*LRFN4* might involve in the immune landscape of LUAD. **(A)** The relative content of 22 immune cell infiltration in LUAD samples. **(B)** The relative content of 8 immune cell infiltration in *LRFN4* ^high^ and *LRFN4* ^low^ groups. **(C–E)** The correlation of *LRFN4* expression with T cells CD4 memory resting Mast cells resting and Macrophages M0. **(F)** The level of ESTIMATEScore, ImmuneScore and TumorPurity in *LRFN4* ^high^ and *LRFN4* ^low^ groups. **(G)** The 6 immune checkpoints (PD-1 (PDCD1), PDL-1 (CD274), PDL-2 (PDCD1LG2), CD86, LAG3, TIGIT) expression in *LRFN4* ^high^ and *LRFN4* ^low^ groups. **(H)** The levels of Tumor Immune Dysfunction and Exclusion (TIDE), Interferon gamma (IFNG), Exclusion, Myeloid-derived suppressor cells (MDSC), and cytotoxic T lymphocytes (CTL). *p < 0.05, **p < 0.01, ***p < 0.001, ****p < 0.0001.

In TCGA-LUAD cohort, we calculated the relative content of 22 immune cell infiltration in LUAD samples using CIBERSORT (Figure 4A). As presented in Figure 4B, the relative contents of 3 immune cells’ infiltration (activated memory CD4+T cells, T follicular helper cells (Tfh), M0 macrophages) were significantly reduced and 5 immune cells’ infiltration (resting memory CD4+T cells, monocytes, resting dendritic cells, activated dendritic cells, resting mast cells) were significantly increased in *LRFN4* ^high^ group compared to in *LRFN4* ^low^ group. Moreover, the levels of CD4+T cell subsets, such as effector memory CD4+T cells, memory CD4+T cells, T helper 1 cell (Th1), and Th2 were significantly different between *LRFN4* ^high^ and *LRFN4* ^low^ groups using xCell algorithm (Supplementary Figure S2A). MCPcounter algorithm showed that Monocyte was also higher in *LRFN4* ^high^ group compared to in *LRFN4* ^low^ group (Supplementary Figure S2B). *LRFN4* expression was significantly negatively correlated with resting memory CD4+T cells and resting mast cells (Figures 4C, D), and was remarkably positively associated with M0 macrophages (Figure 4E).

Moreover, we found that the ESTIMATE Score and Immune Score were observably decreased and Tumor Purity was significantly increased in *LRFN4* ^high^ group compared to in *LRFN4* ^low^ group (Figure 4F). The 6 immune checkpoints (PD-1 (PDCD1), PDL-1 (CD274), PDL-2 (PDCD1LG2), CD86, LAG3, TIGIT) expression were markedly increased in *LRFN4* ^high^ group compared to *LRFN4* ^low^ group (Figure 4G). We further analyzed the TIDE in both the *LRFN4* ^high^ and *LRFN4* ^low^ groups to evaluate the response to immunotherapy. As shown in Figure 4H, TIDE, Interferon gamma (IFNG), Exclusion, Myeloid-derived suppressor cells (MDSC), and cytotoxic T lymphocytes (CTL) were significantly elevated in the *LRFN4* ^high^ group (high vs low, p < 0.05). These results suggested that LUAD patients with high *LRFN4* expression were less likely to benefit from immunotherapy.

### 3.5 LRFN4 could predict the prognosis of LUAD patients

**FIGURE 5:**
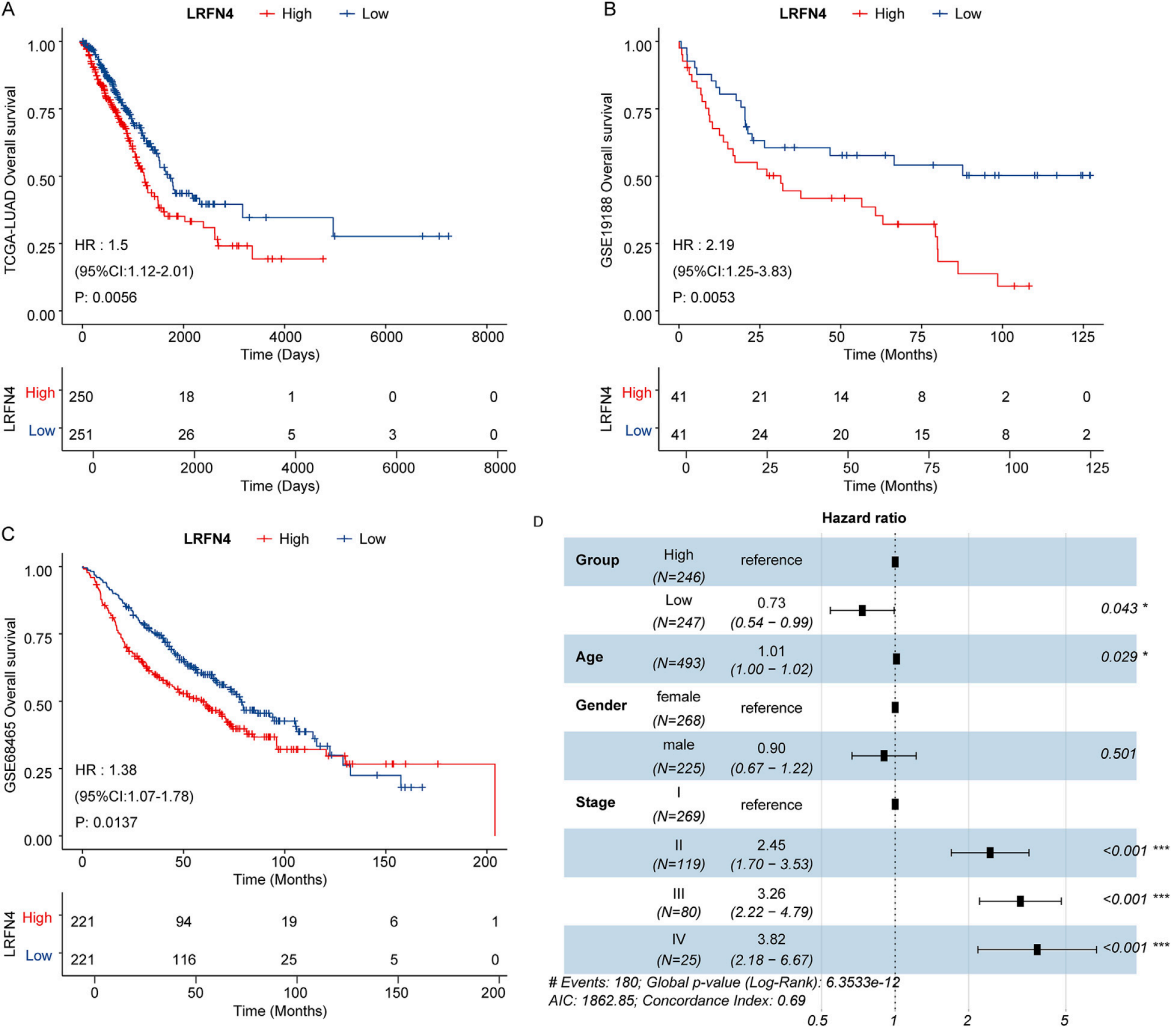
*LRFN4* could predict the prognosis of LUAD patients. **(A)** The Kaplan–Meier survival curve (*LRFN4* ^high^ and *LRFN4* ^low^ LUAD patients) for overall survival in TCGA-LUAD cohort. **(B)** The Kaplan–Meier survival curve (*LRFN4* ^high^ and *LRFN4* ^low^ LUAD patients) for overall survival in GSE19188 dataset. **(C)** The Kaplan–Meier survival curve (*LRFN4* ^high^ and *LRFN4* ^low^ LUAD patients) for overall survival in GSE68465 dataset. **(D)** The multivariate Cox regression analysis including age, gender, stage, *LRFN4* ^high^ and *LRFN4* ^low^ group.

Subsequently, we analyzed the correlation between *LRFN4* expression and prognosis of LUAD patients. In TCGA-LUAD, GSE19188 and GSE68465 cohorts, the patients in *LRFN4* ^high^ group were correlated with inferior prognosis (Figures 5A–C). In TCGA-LUAD cohort, we preformed the multivariate Cox regression analysis to further determine the independent prognostic indicators, including age, gender, stage and *LRFN4* ^high^ and *LRFN4* ^low^ groups (eight samples without stage information were removed). The results showed that *LRFN4* could independently predict the prognosis of LUAD patients (Figure 5D). Furthermore, we examined the relationship between LRFN4 and other biomarkers, specifically *YKT*6 (Zhang et al., 2024a), *SPOCK1* (Liu et al., 2023), and *MCM4* (Tan et al., 2023), in relation to the prognosis of LUAD patients. Our analysis revealed that, compared to the other biomarkers (*YKT6*, *MCM4*), LRFN4 exhibited a stronger correlation with the prognosis of LUAD patients (Supplementary Figure S3).

### 3.6 The copy number variation (CNV), gene mutation and tumor mutation burden (TMB) in LRFN4^high^ and LRFN4^low^ LUAD patients

**FIGURE 6:**
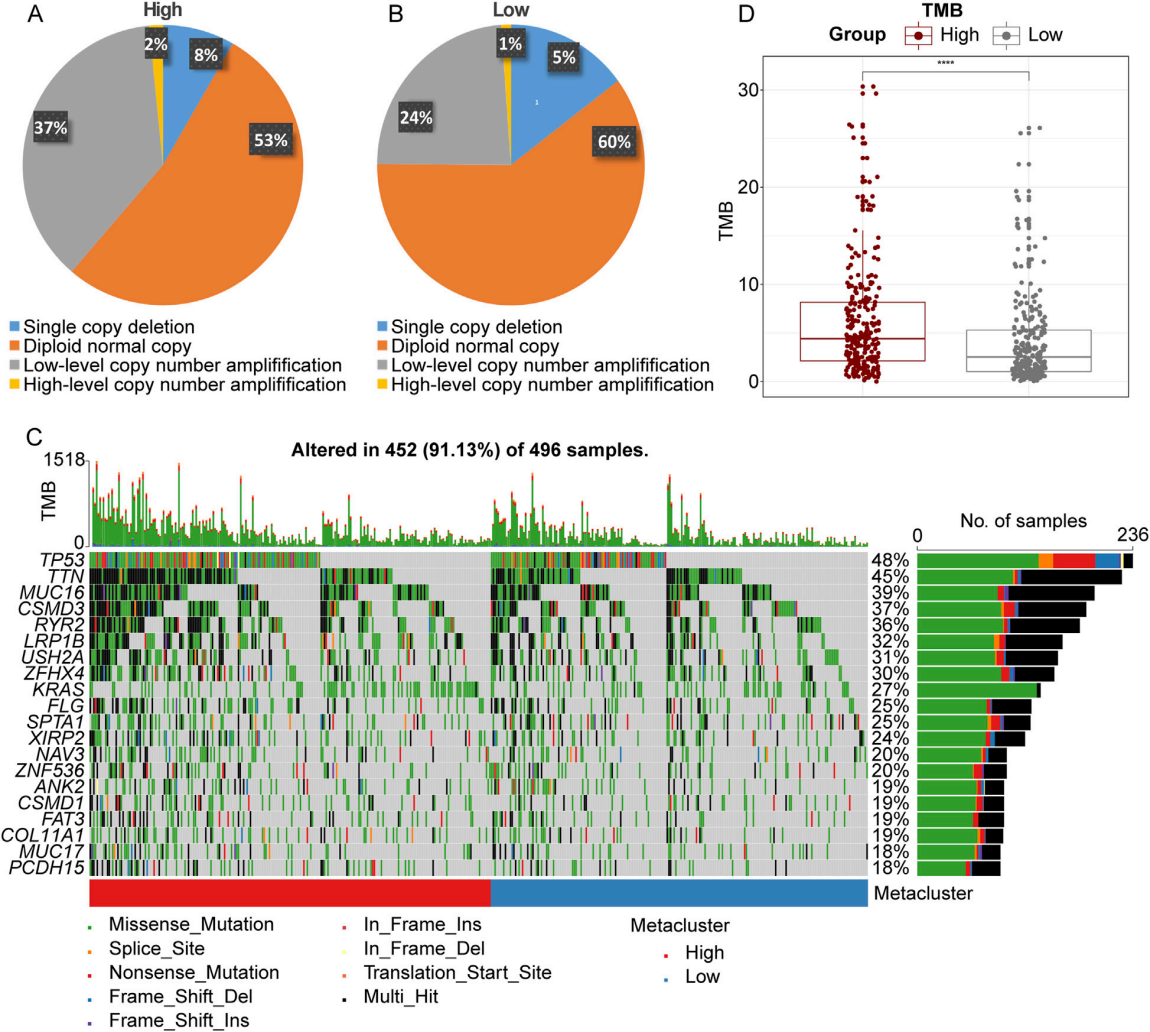
The copy number variation (CNV), gene mutation and tumor mutation burden (TMB) in LRFN4^high^ and LRFN4^low^ LUAD patients. **(A, B)** The CNV in *LRFN4* ^high^ and *LRFN4* ^low^ groups. **(C)** The mutation rate of genes in *LRFN4* ^high^ and *LRFN4* ^low^ groups. **(D)** The level of TMB in *LRFN4* ^high^ and *LRFN4* ^low^ groups.

To investigate the potential cause of upregulation of *LRFN4* in LUAD, we analyzed the CNV in *LRFN4* ^high^ and *LRFN4* ^low^ groups using TCGA-LUAD cohort. As showed in Figures 6A, B, the high-level copy number amplification accounted for 53% and 60% in *LRFN4* ^high^ group and *LRFN4* ^low^ group, respectively. Thus, the copy number amplification might be one of the reasons for *LRFN4* upregulation in LUAD patients. Next, we analyzed the gene mutation and TMB level in *LRFN4* ^high^ and *LRFN4* ^low^ groups. The mutation rate of TP53, TTN and MUC6 were increased in *LRFN4* ^high^ group compared to *LRFN4* ^low^ group (Figure 6C). The level of TMB was observably increased in *LRFN4* ^high^ group compared to *LRFN4* ^low^ group (Figure 6D).

### 3.7 The correlation of TCF3, E2F1, E2F3, LRFN4 expression with drug sensitivity

**FIGURE 7:**
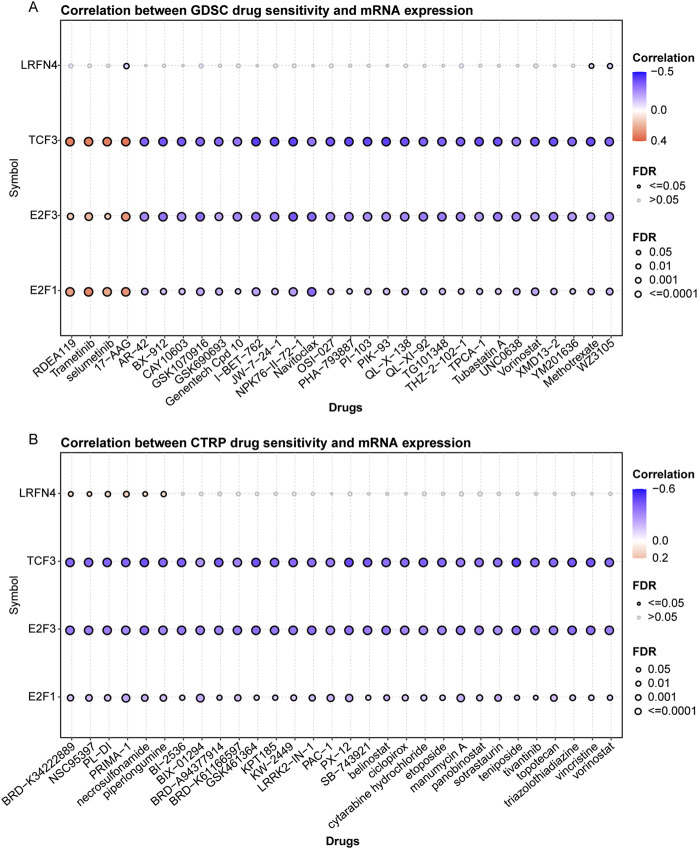
The correlation of TCF3, E2F1, E2F3, *LRFN4* expression with drug sensitivity. **(A, B)** The correlation of TCF3, E2F1, E2F3, *LRFN4* expression with IC50 of drugs in Genomics of Drug Sensitivity in Cancer (GDSC) and Response Portal for Therapeutic Genomics (CTRP).

Finally, we analyzed the correlation of TCF3, E2F1, E2F3, *LRFN4* expression with drug sensitivity. We collected a total of 265 small molecules of IC860 and 481 small molecules of IC1001 from 50 different cell lines, along with their corresponding mRNA gene expressions in the GDSC and CTRP, and combined the mRNA expression and drug sensitivity data. We found that in GDSC, *LRFN4* expression was negatively correlated with IC50 of 17-AAG, the E2F1, E2F3 and TCF3 expression was positively associated with the IC50 of RDEA119, trametinib, selumetinib and 17-AAG, and was negatively correlated with the IC50 of many drugs, such as AR-40, BX-912 (Figure 7A; Supplementary Table S3). In CTRP, *LRFN4* expression exhibited positive correlation with the IC50 of BRD−K34222889, NSC95397, PL-DI, PRIMA-1, necrosulfonamide, while the E2F1, E2F3 and TCF3 expression had negative association with the IC50 of these drugs (Figure 7B; Supplementary Table S4).

## 4 Discussion

Previous studies have reported that the LRFN proteins were essential for neuritis development and branching, as well as synapse formation and maturation (Ko et al., 2006; Lie et al., 2016). In recent years, some researchers have found that in nonneural tissues, the LRFN were also expressed and exerted vital functions. The LRFN2 protein may subvert hematopoietic differentiation to enhance erythropoiesis, and it can cause erythroblastosis in MEnTCD2.5 lymph node cells when it collaborates with Myc (Castellanos et al., 2007). Noteworthily, Konakahara and colleagues have revealed that LRFN4 protein was expressed in a wide range of human cancer and leukemia cell lines, gastric cancer MKN7 cells, T-cell leukemia Jurkat cells, breast cancer SK-BR-3 cells Burkitt’s lymphoma Ramos cells and monocytic leukemia THP-1 and U937 cells (Konakahara et al., 2011). *LRFN4* is a risk gene for ovarian cancer (Li et al., 2021) and a prognostic biomarker for stomach adenocarcinoma (Han et al., 2021). Liu et al. have reported that the in clinical gastric cancer tissues, *LRFN4* expression was elevated in tumor cells and fibroblasts, and the overexpress of *LRFN4* was substantially linked with tumor invasive features, and the gastric cancer patients with high *LRFN4* expression had poor prognosis (Liu et al., 2019a). In the present study, *LRFN4* was high expressed in LUAD patients, and the patients with upregulated *LRFN4* exhibited inferior prognosis. In addition, in CRC, *LRFN4* expression was closely correlated with tumor location, T staging, N staging and TNM staging (Zheng et al., 2020), these findings were consistent with our results. Moreover, we also discovered that *LRFN4* expression was correlated with metastasis and age of LUAD patients. Accordingly, *LRFN4* was a risk gene for LUAD, and it might predict the prognosis of LUAD patients.

In addition, we discovered that the expression of *LRFN4* was regulated via transcription factors E2F1 and E2F3 by binding upstream of *LRFN4* in LUAD. E2F1 and E2F3 are members of E2F family, and this family genes were important in the development of tumors because they could regulate DNA replication and cell cycle progression (Chen et al., 2009; Liu et al., 2015). It has been demonstrated that the level of E2F1 and E2F3 expression were related to the clinical outcomes of multiple tumors, such as lung cancer (Ren et al., 2017), pediatric retinoblastoma (Chen et al., 2022), breast cancer (Han et al., 2020), hepatocellular carcinoma (Han et al., 2019). Sun and colleagues have indicated that the levels of E2F1 and E2F3 expression were increased in LUAD and lung squamous cell carcinoma (LUSC) tissues (Sun et al., 2018). They also found that the high transcription level of E2F1 was linked with shorter relapse-free survival (RFS), while the high E2F3 transcription level was correlated with longer RFS in lung patients (Sun et al., 2018). Wang et al. have revealed that the high expression of E2F1 was significantly related to poor patient survival in lung cancer (Wang et al., 2021). The E2F1 expression exhibited positive associate with Ki-67 proliferation index in NSCLC cells (Meng et al., 2020). Furthermore, in gastric cancer, E2F1 was a potential downstream target of CHPF, and knockdown of E2F1 reduced the CHPF-induced promotion of gastric cancer (Lin et al., 2021a). In pediatric retinoblastoma, E2F1 could increase the expression of CKS2 via binding to its promotor, thereby promoting the promotes cell proliferation and tumor formation (Chen et al., 2022). In endometrial cancer, the knocking down E2F3 could inhibit the ability of HOXB9 in promoting migration of tumor cell (Wan et al., 2018). In this work, the E2F1 and E2F3 had distinct binding peak upstream of *LRFN4* gene in LUAD. These evidences indicated that E2F1 and E2F3 might regulate the expression of *LRFN4* to involve in the progression of LUAD. However, this requires further verification, and it would be an interesting question for a future study.

We also discovered that *LRFN4* expression was significantly negatively correlated with T cells CD4 memory resting and Mast cells resting, and was remarkably positively associated with Macrophages M0. The T cells predominated in lung cancer immune landscape, and CD4^+^ T cells and CD8^+^ T cells were the common prevalent T cell subtypes (Kraemer et al., 2023). The increased levels of CD3^+^ and CD8+T cells exhibited better prognosis of NSCLC patients (Schalper et al., 2015), and the NSCLC patients with higher CD8^+^ counts had longer overall survival rate. It has been reported that the inactivated mast cells and inactivated CD4 memory T cells were closely associated with good prognosis of LUAD patients (Gentles et al., 2015). Mast cells are innate immune cells that live in the tissue and play an important role in the inflammatory response and tissue homeostasis (Plum et al., 2024; Shu et al., 2025). In tumor microenvironment, mast cells could regulate cell proliferation and survival, invasiveness, metastasis, and angiogenesis (Aponte-Lopez and Munoz-Cruz, 2020). M1 macrophages limit tumor growth by secreting proinflammatory cytokines such as IL-6, IL-8, and TNF-α (Zhang et al., 2024b; Hsiao et al., 2025; Murray et al., 2014). The M2 macrophages promoted the PD-1 expression, inhibiting T cell immune function and promoting tumor cell immune escape (Gordon et al., 2017). Konakahara et al. have found that in the monocytic cell line THP-1 and in primary monocytes, *LRFN4* expression is elevated following macrophage differentiation (Konakahara et al., 2011). Based on this evidence, *LRFN4* may promote an immunosuppressive environment in LUAD, potentially impeding T cell activity and diminishing the effectiveness of treatments such as immune checkpoint inhibitors (ICIs). Immune checkpoints, including PD-1, play a crucial role in regulating T cell responses (Andrews et al., 2024). In the present study, PD-1 expression was significantly increased in the *LRFN4* high-expression group compared to the *LRFN4* low-expression group. This indicates that *LRFN4* may promote T cell exhaustion by upregulating PD-1 expression, thereby inhibiting anti-tumor immune responses.

Furthermore, the level of TMB was notably higher in the *LRFN4* high-expression group compared to the *LRFN4* low-expression group. TMB has been associated with improved responses to immunotherapy in various cancers (Ribas and Wolchok, 2018) and is positively correlated with the efficacy of PD-1 checkpoint inhibition (Goodman et al., 2017). However, we observed that patients with LUAD exhibiting elevated levels of LRFN4 expression had higher TIDE scores, suggesting that these patients may be less likely to benefit from immunotherapy. These results indicate that although TMB levels were elevated in the high-expression group of *LRFN4*, it is plausible that *LRFN4* inhibits T cell activity through the upregulation of PD-1 and PD-L1 expression, which may consequently reduce the efficacy of ICIs. Moreover, drug sensitivity showed that *LRFN4* and transcription factors (E2F1 and E2F3) were correlated with IC50 of multiple drugs, indicating that the expression of *LRFN4* and the transcription factors that regulate its expression in LUAD may be of great significance in guiding clinical medication.

While this study elucidates the expression of *LRFN4* in LUAD and its implications for prognosis, tumor microenvironment, and drug sensitivity, several limitations warrant attention. First, the study is based on retrospective data from TCGA and GEO database, the limited sample size in public databases may cause possible bias to the results, and lacks validation through independent clinical samples. Thus, prospective studies with larger sample sizes are essential for future research. Additionally, although GSEA has identified potential pathways involving *LRFN4* in LUAD, further functional experiments are necessary to clarify the precise mechanisms by which *LRFN4* contributes to LUAD progression. Furthermore, the predicted interactions of transcription factors with the *LRFN4* promoter require validation through experimental methods. Lastly, a comprehensive understanding of the role of *LRFN4* in the LUAD prognosis, immune microenvironment and its implications for immunotherapy necessitates additional clinical samples and prospective studies.

## 5 Conclusion

*LRFN4* is significantly upregulated in LUAD samples and cells and is correlated with poor prognosis. As a new prognostic biomarker for LUAD, *LRFN4* may be a potential therapeutic target for LUAD patients. *LRFN4* is associated with immunological characteristics of LUAD. This study provides new insights into the prediction of disease progression and the development of targeted therapies for LUAD. However, the biological role of *LRFN4* in the prognosis and immune microenvironment of LUAD patient needs to be further studied in the future.

## Data Availability

The original contributions presented in the study are publicly available. This data can be found in The Cancer Genome Atlas (TCGA) database at [https://tcga-data.nci.nih.gov/tcga/] and the Gene Expression Omnibus (GEO) database at [https://www.ncbi.nlm.nih.gov/geo/] (accession number: GSE116959, GSE19188 and GSE68465).

## Supplementary material

SUPPLEMENTARY FIGURE S1 The correlation between LRFN4 expression and gender.

SUPPLEMENTARY FIGURE S2 The immune cell infiltration of LUAD was estimated via xCell **(A)** and MCPcounter **(B)** algorithms. *p < 0.05, **p < 0.01, ***p < 0.001, ****p < 0.0001.

SUPPLEMENTARY FIGURE S3 The result of univariate Cox regression analysis.

## Abbreviations

LUAD, lung adenocarcinoma; CNV, copy number variation; TMB, tumor mutation burden; AIS, adenocarcinoma *in situ*; MIA, minimally invasive adenocarcinoma; LRFN, Leucine rich repeat and fibronectin; SALM, synaptic adhesion like molecules; LRR, leucinerich repeat; CRC, colorectal cancer; TCGA, The Cancer Genome Atlas; GEO, Gene Expression Omnibus; DEGs, differentially expressed genes; GSEA, Gene set enrichment analysis; GDSC, Genomics of Drug Sensitivity in Cancer; TCF3, transcription factors; CNV, copy number variation; TMB, tumor mutation burden; LUSC, lung squamous cell carcinoma; RFS, relapse-free survival.
