# Supplementary figures and images for: Significance of LRFN4 in prognosis and tumor microenvironment of lung adenocarcinoma
Source: Front Pharmacol. 2025 Feb 25;16:1540636. doi: 10.3389/fphar.2025.1540636 (PMC11893870; doi:10.3389/fphar.2025.1540636)

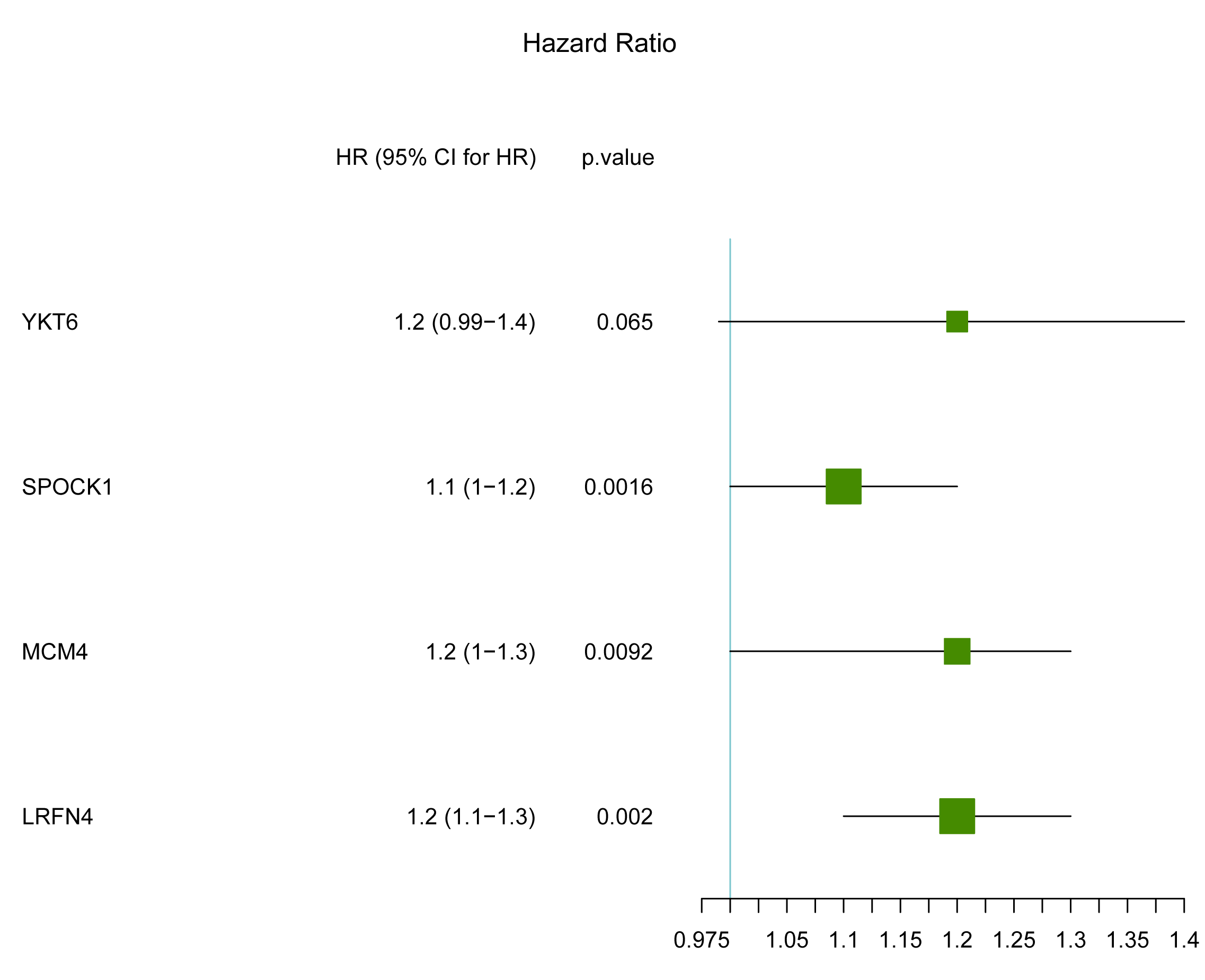

Supplement: Supplementary file 2 [file Image3.tif]

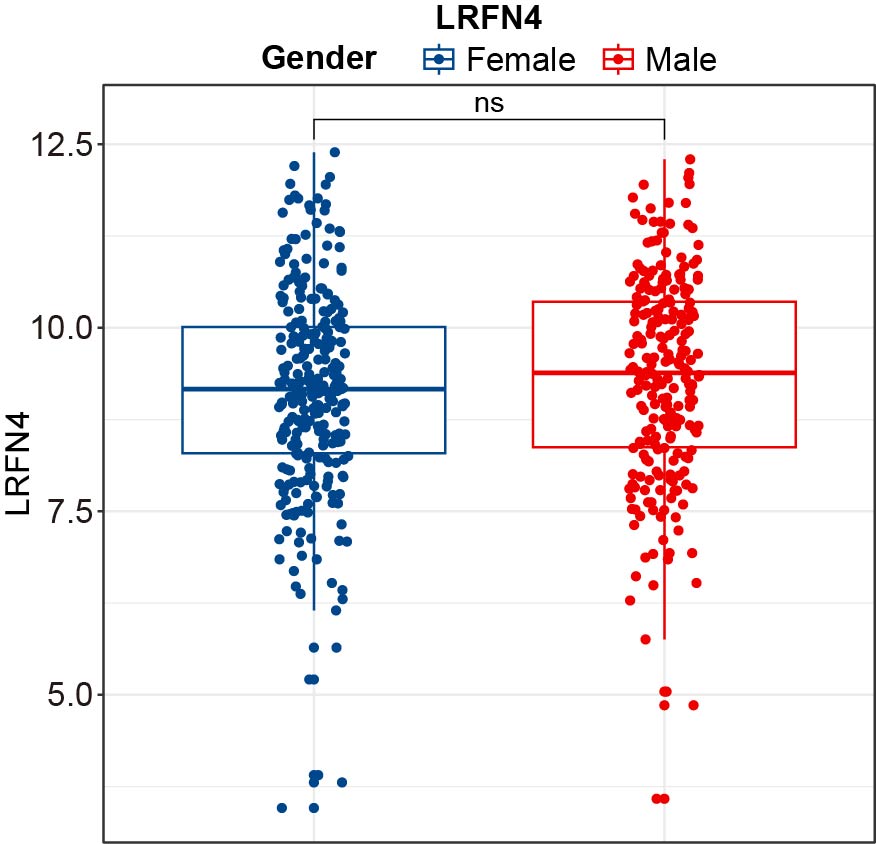

Supplement: Supplementary file 3 [file Image1.jpeg]

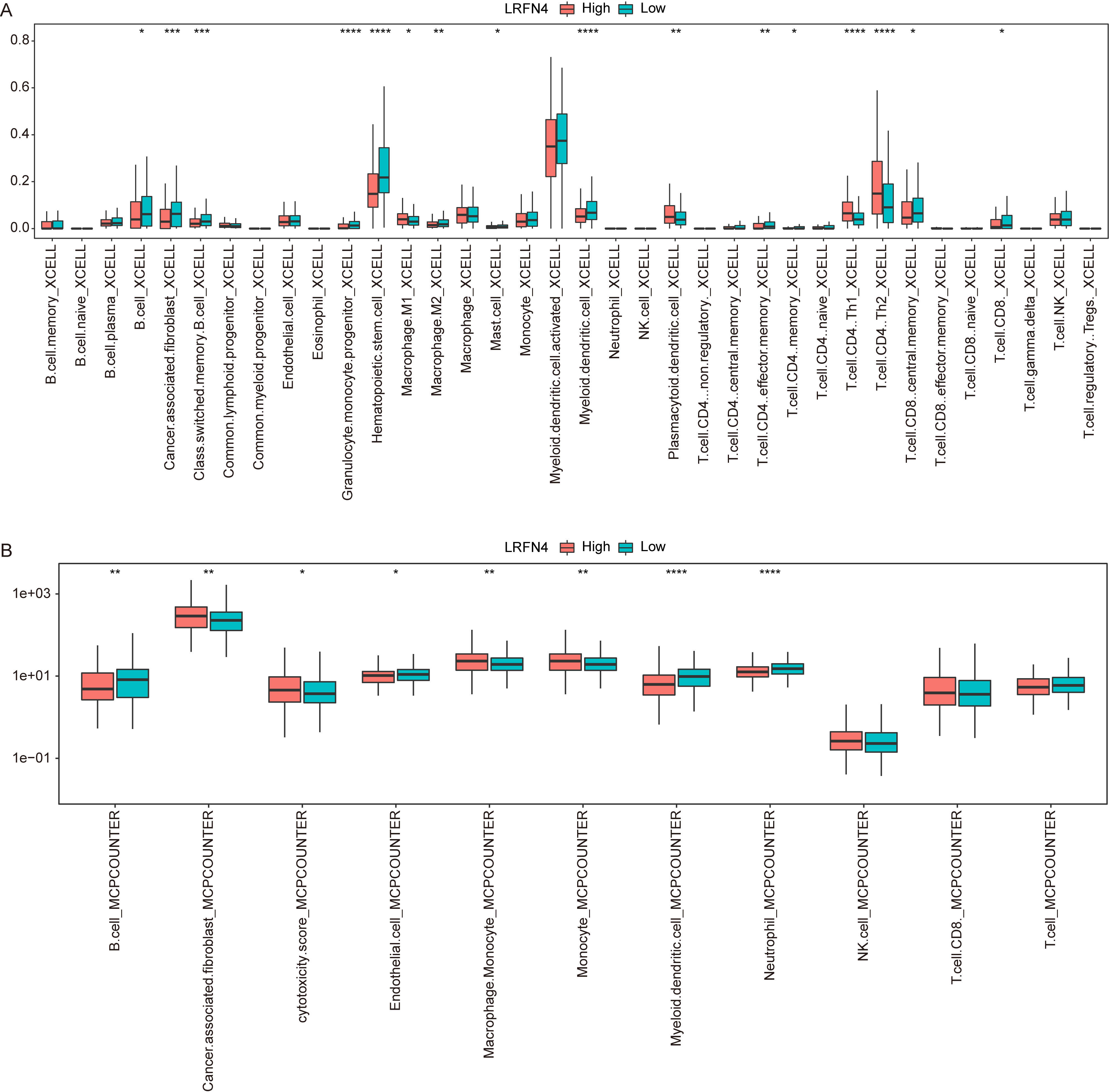

Supplement: Supplementary file 4 [file Image2.tif]
